# Does Childhood Obesity Trigger Neuroinflammation?

**DOI:** 10.3390/biomedicines10081953

**Published:** 2022-08-11

**Authors:** Valeria Domenica Zingale, Simone D’Angiolini, Luigi Chiricosta, Valeria Calcaterra, Giorgio Giuseppe Orlando Selvaggio, Gianvincenzo Zuccotti, Francesca Destro, Gloria Pelizzo, Emanuela Mazzon

**Affiliations:** 1 IRCCS Centro Neurolesi “Bonino-Pulejo”, Via Provinciale Palermo, Contrada Casazza, 98124 Messina, Italy; 2 Department of Internal Medicine, University of Pavia, 27100 Pavia, Italy; 3 Pediatric Department, “V. Buzzi” Children’s Hospital, 20154 Milan, Italy; 4 Pediatric Surgery Department, “V. Buzzi” Children’s Hospital, 20154 Milan, Italy; 5 Department of Biomedical and Clinical Science, University of Milan, 20157 Milan, Italy

**Keywords:** childhood obesity, inflammatory response, neuroinflammation, metabolism, transcriptomic analysis

## Abstract

Childhood obesity is constantly increasing around the world, and it has become a major public health issue. Considerable evidence indicates that overweight and obesity are important risk factors for the development of comorbidities such as cognitive decline, neuroinflammation and neurodegenerative diseases. It is known that during obesity, adipose tissue undergoes immune, metabolic and functional changes which could induce a neuroinflammatory response of the central nervous system (CNS). In this context, to inspect if obesity can start to trigger the neuroinflammation from a pediatric age, we surgically collected and analyzed adipose tissue from the periumbilical area of three obese children (AT-OB) and two normal-weight children (AT-Ctrl). We considered the transcriptomic profile of our samples to detect alterations in different biological processes that might be also involved in the inflammatory and neuroinflammatory response. Our results show alterations of lipid and fatty acids metabolism in AT-OB compared to the AT-Ctrl. We also observed an onset of inflammatory response in AT-OB. Interestingly, among the genes involved in neuroinflammation, *GRN* and *SMO* were upregulated, while *IFNGR1* and *SNCA* were downregulated. Our study highlights that obesity may trigger inflammation and neuroinflammation from a pediatric age.

## 1. Introduction

The increasing incidence of obesity has become a significant public health concern [1]. Moreover, the World Health Organization (WHO) estimated that more than 42 million children under the age of 5 are overweight or obese [2]. These conditions increase the risk of developing comorbidities such as type 2 diabetes mellitus, atherosclerosis, myocardial infarction and stroke [3]. In addition, emerging evidence links obesity with increased cognitive decline and the development of neurodegenerative diseases [4].

Childhood obesity is associated with an increased incidence of overweight in adults and an increased risk of developing obesity-related comorbidities contributing to premature mortality in adulthood [5]. Obesity has already been inspected in in vivo experiments. In this line, transcriptomic inspections on different species confirmed that abnormal changes in gene expression level in a variety of tissues are closely linked to metabolic problems derived from the obesity condition [6,7].

Obesity is associated with the development of chronic inflammation in adipose tissue, which gradually becomes systemic and can induce a neuroinflammatory response of the central nervous system (CNS) [8]. Several findings shows that obesity and a high-fat diet can lead to cognitive dysfunctions, causing systemic inflammation and an excess of circulating free fatty acids (FFA) [9].

In fact, obesity leads to increased levels of circulating free fatty acids, pro-inflammatory cytokines, chemokines and immune cells, which in turn gain access to the hypothalamus by increasing the permeability of the blood–brain barrier (BBB) [10]. This determines central inflammation that causes synaptic remodeling, neuronal apoptosis, and altered neurogenesis. Together, these mechanisms can lead to long-term changes in cellular signalling and connectivity, even causing neurodegeneration and brain atrophy [9].

To identify the different signals related to inflammatory and dysmetabolic process, we explored the transcriptomic profile of adipose tissue (AT) analyzing differences between obese children (AT-OB) and a control group (AT-Ctrl). We aimed to evaluate transcriptionally whether the initiating events of dysmetabolism and inflammation related to obesity start early in childhood. Since the evidence indicates that obesity has a negative impact on brain function in adulthood and that it may result in neuroinflammation, it is important to assess its impact during childhood and adolescence.

## 2. Materials and Methods

### 2.1. Patients

We gathered 5 adipose tissue samples surgically collected from 2 normal-weight children (males, aged 9 years) (AT-Ctrl) and 3 obese children (2 males aged 13 and 10 years, respectively, along with a female aged 14 years) (AT-OB). The body mass index (BMI) of AT-Ctrl was lower than the 75th percentile, whereas the BMI of AT-OB exceeded the 95th percentile. In order to perform a comparable analysis between the different samples, we collected the adipose tissue from the same area for each patient. Specifically, adipose tissue was collected from the periumbilical area. The institutional ethics committee approved the study (MI area 1–12/2016/2020), and it was conducted in accordance with the Helsinki Declaration of 1975, as revised in 2008. Table 1 reports the clinical features of AT-Ctrl and AT-OB, along with metabolic characterization [11,12].

### 2.2. RNA Extraction

RNA isolation from adipose tissue was performed using the TRIzol Reagent method (ThermoFisher Scientific, Rockford, IL, USA). The tissues were disrupted using a syringe/needle. About 100 mg of samples were introduced in a 5 mL RNase DNase free tube containing 1 mL of TRIzol. Homogenization was performed by several back and forth movements through a syringe and needle (18 G) [13]. After homogenization, the samples were incubated at room temperature for 5 min to permit complete dissociation of the nucleoproteins complex. Subsequently, a centrifugation step at 12,000× *g* at 4 °C for 5 min was performed, and the resulting fat monolayer was carefully avoided while the underlying light layer was transferred into a clean 1.5 mL tube. Then, 200 µL of chloroform was added to the sample and stirred for 15 s and mixed by shaking. After 10 min of incubation, the samples were centrifuged at 12,000× *g* at 4 °C for 15 min. After centrifugation, the samples separate into three phases, a lower red phenol-chloroform, an interphase, and a colorless upper aqueous phase. The aqueous phase containing RNA was transferred to a new tube and 500 µL of isopropanol was added. After 10 min of incubation, the samples were centrifuged at 12,000× *g* at 4 °C for 10 min. The supernatant was discarded and the pellet was resuspended in 1 mL of 75% ethanol and mixed by shaking. Then, the samples were centrifuged at 7500× *g* at 4 °C for 5 min, the supernatant was discarded, and the pellet was left to air dry for 10 min. Finally, the pellet was resuspended in 30 µL of RNase-free water.

### 2.3. RNA Library Preparation

The RNA quality and concentration were measured using Eppendorf BioSpectrometer fluorescence. For the preparation of the library, TruSeq RNA Exome protocol (Illumina, San Diego, CA, USA) was used. The library was analyzed using the Illumina instrument Miseq.

The first strand of cDNA was synthesized using the SuperScript II Reverse Transcriptase (Invitrogen, Milan, Italy), and after, the Second Strand Marking Master Mix was used to obtain the double strand of the cDNA. The reaction mix was purified using AMPure XP beads (Beckman Coulter, Brea, CA, USA). Fragment adenylation was performed at the 3’ ends to bind complementary adapters-indices to the cDNA fragment. The cDNA fragments were amplified by PCR. The DNA libraries were pooled together using 200 ng for each one, and two hybridization cycles were performed. The purification of the pool was obtained by streptavidin conjugated magnetic beads, and then a second PCR amplification was conducted. Finally, the libraries were purified using the AMPure XP.

### 2.4. Bioinformatics Analysis

To filter out the reads with low qualities and remove the adapters, we used Trimmomatic v.0.40-rc1 (Usadel Lab, Aachen, Germany) [14]. After the trimming phase, we aligned the reads to a reference genome using Spliced Transcripts Alignment to a Reference (STAR) RNA-seq aligner v.2.7.10a_alpha_220207 (New York, NY, USA) [15]. The genome used as reference during the alignment phase was the human reference genome GRCh38 v.39. We used htseq-count (European Molecular Biology Laboratory (EMBL), Heidelberg, Germany) [15] to obtain the transcriptional counts from our aligned reads. Using the programming language R (R Core Team) and through the package limma [16], specific for analysis of the different expression of microarray data, we conducted a statistical analysis of the differentially expressed genes (DEGs). To filter the DEGs, we used the q-value. We left only those that had a q-value lower than 0.05. The q-value was calculated from the p-value using the false discovery rate method to limit the number of false positives. To inspect different gene ontologies, we used the GO database [17]. To explore the resulting DEGs and observe if these were involved in specific biological processes, we used Amigo2 [18]. We obtained the enriched ontologies using tools integrated in the PANTHER database [19].

## 3. Results

Analysis of raw data revealed the comparability among the samples; results are shown in Table S1. Additionally, we focused our attention on the distance of transcriptomic profiles of each sample for each group. The principal component analysis in Figure S1 highlights that the first component is able to discriminate AT-OB and AT-Ctrl with a variance of 81%. The comparison between obese and normal-weight children resulted in 932 DEGs. Among these DEGs, 498 were upregulated in AT-OB (Table S2) and 434 were downregulated in AT-OB (Table S3). We inspected, using the AmiGO 2 database, the biological processes “Fatty acid metabolic process” (GO:0006631), “Lipid metabolic process” (GO:0006629) and “Carbohydrate metabolic process” (GO:0005975). We chose to analyse these biological processes to whether they were altered in the obesity conditions of our samples. In Table 2, we can see which genes are involved in the “Fatty acid metabolic process”, “Lipid metabolic process” and “Carbohydrate metabolic process” that were also DEGs in our analysis.

In Table 2, we can see that 19 DEGs are involved in the “Fatty acid metabolic process”, 7 of which are upregulated and 12 are downregulated. Moving to the “Lipid metabolic process”, we observed 59 DEGs involved, and among these 32 are upregulated and 27 are downregulated. The last biological process taken into account was the “Carbohydrate metabolic process”, for which we observed nine DEGs involved, and among these, six were upregulated and three were downregulated. To have a more precise overview of these biological processes, we inspected the genes that were DEGs that act as positive or negative regulators.

In Table 3, we can see all the DEGs that were positive and negative regulators for the biological processes previously analysed. In the “Regulation of fatty acid metabolic process” (GO:0019217), we observed two DEGs, one upregulated and one downregulated, that act as positive regulators, and three DEGs, all downregulated, that act as negative regulators of this process. Regarding the “Regulation of lipid metabolic process” (GO:0019216), there were eight DEGs that act as positive regulators, four upregulated and four downregulated, and seven that act as negative regulators of the process. Among these, one was upregulated and six were downregulated. In the “Regulation of carbohydrate metabolic process” (GO:0006109), seven DEGs, all downregulated, acted as positive regulators, and three acted as negative regulators, two of which were upregulated, and one was downregulated.

Considering the correlation among inflammatory response and overweight condition, we chose to also inspect the process related to the “Inflammatory response” (GO:0006954) and “Regulation of inflammatory response” (GO:0050727), and determine whether some DEGs are involved in it. The results related to “Inflammatory response” and its regulators are reported in Table 4.

**Figure 1:**
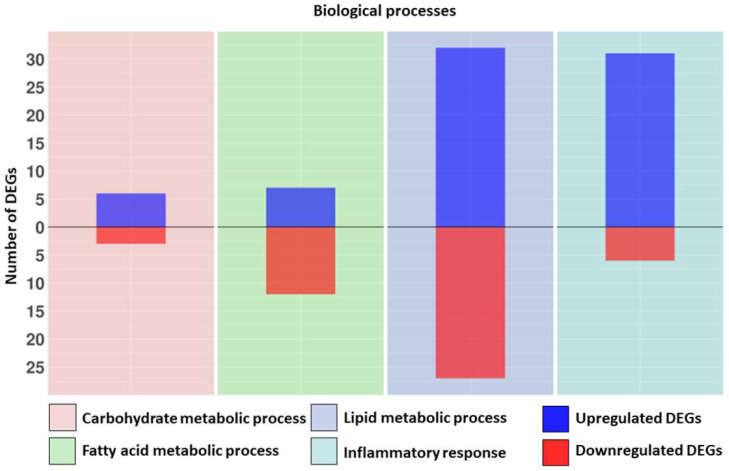
Bar plot distributions of Differentially expressed genes (DEGs) related to the “Carbohydrate metabolic process”, “Fatty acid metabolic process”, “Lipid metabolic process” and “Inflammatory response”. Blue indicates the upregulated DEGs, and red indicates the downregulated DEGs for each biological process. The figure is split into four sections, one for each biological process.

In total, 37 DEGs are involved in the “Inflammatory response”, among which 31 are upregulated and 6 are downregulated. We found 11 genes related to positive regulation of this process, 8 upregulated and 3 downregulated. Eight DEGs are involved in negative regulation of the inflammatory response, among which five are upregulated and three are downregulated. We checked if among the DEGs reported in Table 4 some were specific to the “Neuroinflammatory response” (GO:0150076). Among the DEGs involved in the “Inflammatory response” *GRN*, *IFNGR1*, *SMO* and *SNCA* were specific to the “Neuroinflammatory response”. The only positive regulator presented in Table 4 also involved in “Regulation of neuroinflammatory response” (GO:0150077) is *PLCG2*, and the negative regulators are *GRN* and *IGF1.* In Figure 1, the distributions of the DEGs related to the “Carbohydrate metabolic process” (Table 2), “Fatty acid metabolic process” (Table 2), “Lipid metabolic process” (Table 2) and “Inflammatory response” (Table 4) are reported.

**Figure 2:**
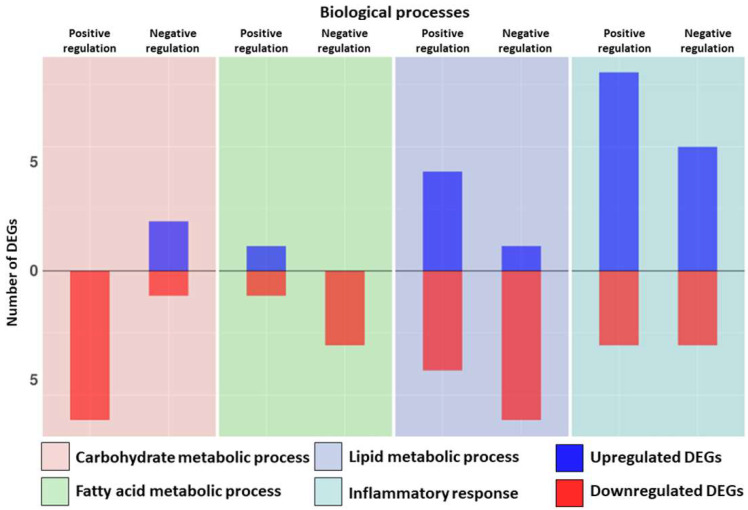
Bar plot distributions of Differentially expressed genes (DEGs) related to the “Regulation of carbohydrate metabolic process”, “Regulation of fatty acid metabolic process”, “Regulation of lipid metabolic process” and “Regulation of inflammatory response”. Blue indicates the upregulated DEGs and red indicates the downregulated DEGs for each biological process. The figure is split into four sections, one for each biological process. For each section, the distributions of DEGs that act as positive or negative regulators for that biological process are reported.

In Figure 2, the distributions of the DEGs related to the “Regulation of Carbohydrate metabolic process” (Table 3), “Regulation of fatty acid metabolic process” (Table 3), “Regulation of lipid metabolic process” (Table 3) and “Regulation of inflammatory response” (Table 4) are reported.

## 4. Discussion

Childhood obesity is increasing all over the world, and it has become a serious challenge for global public health [20]. It is known that during obesity, adipose tissue undergoes immune, metabolic and functional changes [4]. The inflammatory environment in adipose tissue is also determined by the production of FFA from the increased lipolytic process of hypertrophic adipocytes. [21]. Dysfunctions in carbohydrates and lipid metabolism and systemic inflammation associated with obesity could induce a neuroinflammatory response of the CNS. Cytokine production and excess of FFA can reach the hypothalamus and determine a local inflammation, which could result in synaptic alterations and neurodegeneration as a manifestation of accelerated aging related to inflammation [9].

We explored the transcriptomic profile of AT collected from pediatric patients, analyzing differences between AT-Ctrl and AT-OB to inspect if obesity can trigger inflammation and neuroinflammation from a pediatric age. In particular, the adipose tissue was collected from the periumbilical area of two AT-Ctrl and three AT-OB.

The obesity condition is known to cause imbalance in metabolic processes [22]. In line with the literature, we first inspected the effects of obesity on lipid, carbohydrates and fatty acids metabolism. Thus, we investigated genes that were included in the GO processes “Lipid metabolic process”, “Carbohydrates metabolic process” and “Fatty acid metabolic process” that were differentially expressed in our analysis.

Additionally, an increase in the expression of the genes involved in the metabolism of fatty acids and lipids and a decrease in carbohydrate metabolism were observed.

Dysfunctional lipid metabolism is the basis of the development of obesity and of complications related to this condition. Mitochondrial fatty acid β-oxidation is the major pathway for the catabolism of fatty acids [23], and appears to be altered in obese children. In our analysis, *CPT1A* and *ACSF3* were upregulated in AT-OB compared to AT-Ctrl, while *HADHA, ACAT1, ACSL1* and *SIRT4* were downregulated. CPT1A (carnitine palmitoyltransferase A1) plays an important role in the transport of long-chain fatty acids into the mitochondria for oxidation, and it is considered the key speed limiting enzyme that catalyzes the conversion of acyl-coenzyme A (acyl-CoA) into acyl-carnitine [24]. ACSF3 (acyl-CoA synthetase family member 3) is a mitochondrial malonyl-CoA synthetase and plays an important role in the synthesis of mitochondrial fatty acids [25]. HADHA (hydroxyacyl-CoA dehydrogenase trifunctional multienzyme complex subunit alpha) catalyzes the last three steps of mitochondrial beta-oxidation of long chain fatty acids, and a reduction in expression was observed in obese people [26]. ACAT1 (acetyl-CoA acetyltransferase 1) is one of the enzymes that catalyzes the last step of the mitochondrial beta-oxidation pathway and mediates the reversible conversion of two molecules of acetyl-CoA to acetoacetyl-CoA [27]. ACSL1 (Acyl-CoA synthetase-1) catalyzes the first activation step of the long-chain fatty acid metabolism [28]. It has been observed that the ACSL1 deficit in the adipose tissue of mice increased adipocytes size and severely impaired fatty acid oxidation [29]. SIRT4 (Sirtuin-4) is a regulator of fatty acid metabolism. It was seen that SIRT4 deficiency leads to increased beta-oxidation, and thus increased transcription of fatty acid oxidation genes [30]. These results show an abnormal oxidation of fatty acids and alteration of lipid metabolism in adipose tissue of obese children compared to the control group.

Moreover, *INSIG-1* and *PRKAA2* are downregulated in AT-OB compared to AT-Ctrl. INSIG-1 (Insulin-induced gene 1) is a key regulatory factor that maintains homeostasis of intracellular lipid metabolism; it suppresses adipogenesis and inhibits the differentiation of preadipocytes to prevent the onset of obesity [31]. PRKAA2 (protein kinase AMP-activated catalytic subunit alpha 2) is a catalytic subunit of the AMP-activated protein kinase (AMPK). AMPK plays a fundamental role in the regulation of fatty acid metabolism, thermogenesis and the development of adipose tissue [32]. One study found that the lack of PRKAA2 subunits may be a contributing factor to the development of obesity. Indeed, mice knockout for PRKAA2 showed an increase in weight and fat mass with increased adipocytes size and accumulation of triglycerides [33]. The downregulation of these genes could determine the activation of the differentiation of the preadipocytes, cause an increase in the number and size of adipocytes, and accumulation of triglycerides and fatty acids, resulting in an increase in fat mass.

In order to investigate the activation of inflammatory processes in childhood obesity, we also focused on the GO processes “Inflammatory response”, “Regulation of inflammatory response” and “Neuroinflammatory response”. Even in this case, an alteration of DEGs involved in inflammation was highlighted.

Among them, *IGF1*, encoding insulin-like growth factor-1, is involved in growth, development, cell differentiation and metabolism, and it has anti-inflammatory activity. Although its role is still unclear in adipose tissue, it has been seen that IGF1 signaling is implicated in reducing glucose levels [34]. Moreover, evidence shows that IGF-I attenuates the inflammatory response induced by FFA accumulation and reduces the expression of pro-inflammatory cytokines [35]. In our analysis, *IGF1* is downregulated in AT-OB, suggesting a reduction of anti-inflammatory activity and increased glucose levels. A reduction of *IGF1* expression in adipose tissue has been shown in obesity conditions [36], and severely obese patients exhibited a low IGF1 associated with significantly higher fat mass and waist circumference [37].

In adipose tissue, PPARG (peroxisome proliferator-activated receptor gamma) stimulates the absorption and accumulation of circulating lipids, resulting in a reduction in circulating levels of FFA [38]. In addition, PPARG plays an important role in the inflammatory response. Numerous studies have also shown that PPARG is able to reduce macrophage infiltration in adipose tissue and chronic inflammation characteristic of obesity. The anti-inflammatory action of PPARG is due to inhibition of nuclear factor κB (NF-kB), resulting in a reduction in the production of cytokines [39]. PPARG activation in adipocytes stimulates a wide array of genes involved in fatty acids uptake including acyl-CoA synthase (ACSL) [40]. In our analysis, *PPARG* is downregulated in AT-OB, and this may result in increased levels of free fatty acids and a reduction of anti-inflammatory activity.

FFA are involved in inflammatory processes through the activation of the Toll-like receptor family (TLR) [21] and by activation of IκB kinases (IKK) [41]. In our analysis, *CD14* and *IKBKB* were overexpressed in AT-OB compared to AT-CTRL. CD14 is a glycoprotein involved in the activation of TLR4 [42]. It has been shown that the expression and activity of CD14 is increased in obesity, metabolic syndrome and diabetes [43]. IKBKB (inhibitor of nuclear factor kappa-B kinase subunit beta) phosphorylates the inhibitor in the inhibitor/NF-kappa-B complex, and it is necessary for the activation of the NF-κB. [44]. NF-κB participates in the induction of numerous immunoregulatory genes whose products include pro-inflammatory cytokines, growth factors, chemokines, adhesion molecules and enzymes that produce secondary inflammatory mediators [45]. Ajuwon et al. demonstrated that FFA can promote inflammation by activating NF-κB, resulting in the synthesis and secretion of chemokines such as chemoattractive monocyte-1 protein (MCP1) from adipocytes, leading to infiltration of proinflammatory macrophages [46].

In addition, *FFAR3* (free fatty acid receptor 3) and *NLRP1* (NLR family pyrin domain containing 1) are upregulated in AT-OB compared to AT-Ctrl. FFAR3 is a G protein-coupled receptor that is activated by a major product of dietary fiber digestion, the short chain fatty acids (SCFAs), plays a role in the regulation of whole-body energy homeostasis, and it may mediate the activation of the inflammatory and immune response [47]. NLRP1 acts as the sensor component of the NLRP1 inflammasome. When activated, NLRP1 inflammasome formation leads to inflammation through the production of proinflammatory cytokines and regulates cell death processes, termed pyroptosis [48]. Pyroptosis is an inflammatory type of programmed cell death characterized by plasma membrane rupture, water influx, cellular swelling, osmotic lysis and release of proinflammatory cytokines into the extracellular space [49]. Studies show that, in obesity, the death of adipocytes is described as death similar to pyrosis [50], where infiltrated macrophages aggregate around each dead adipocyte creating a “crown-shaped” structure and this leads to low-grade chronic inflammation [51].

Finally, in our analysis, among the DEGs involved in the “Inflammatory response”, *GRN*, *IFNGR1*, *SMO* and *SNCA* are specific to the “Neuroinflammatory response”. In particular, *GRN* and *SMO* are upregulated in AT-OB compared to AT-Ctrl.

GRN (granulin precursor) is part of the pro-inflammatory adipokines produced by adipose tissue and plays an important role during the process of inflammation [52]. In addition to being involved in the chronic inflammation associated with obesity, it is also implicated in various pathological states, including neurodegenerative diseases [53]. SMO (smoothened, frizzled class receptor) is a receptor for hedgehog signaling (Hh signaling) proteins. Hh signaling plays a role in lipid metabolism, insulin sensitivity and inflammatory response [54]. Moreover, Hh signaling has recently been implicated in the regulation of adipose tissue, and several studies have shown a correlation between the increase in its expression and the increase in body weight and fat mass [55]. It has already been shown that the expression of SMO ligand was over-regulated in the fat of obese mice, resulting in a reduction in PPARG activity [56], as also evidenced by our results. Hh signaling is related to cellular metabolism and participates in the molecular mechanisms of neurogenesis, promoting synaptic plasticity. Although the involvement of Hh signaling has been reported in many neurological damages, studies on the relationship between Hh signaling and cognitive deficits in obesity are lacking [57]. In light of the above, observing the discussed DEGs and their expression, we can speculate the activation of different types of inflammatory response. Increased levels of expression of FFA, chemokines and proinflammatory cytokines lead us to hypothesize an imbalance in hypothalamic activity that, as previously reported, results in alteration of synaptic remodeling, neural apoptosis and neurogenesis.

It is known that, in the adipose tissue, gender could be a factor that influence genes expression. Indeed, alteration of expression levels derived from gender differences are known to impact immune response or lipid metabolism [58]. In this line, before considering whether including a female sample in our cohort was better than reducing the size of our cohort by removing it, we performed a PCA inspection. The PCA in Figure S1 actually shows that, even with some difference, the transcriptomic profile of the female sample was quite close to the profile of the male samples of the AT-OB group that, interestingly, were all clustered together quite far away from the AT-Ctrl. Indeed, the first component of the PCA is able to discard the two groups with a variance of 81%. In line with our observation and considering the difficulty to obtain adipose tissue from children, we believed that including one more sample to the cohort was more beneficial than reducing the size of the cohort, even if some differences could be present.

Our analysis gives an overview of different mechanisms that can play a critical role not only in the relationship between obesity and neuroinflammation, already widely acknowledged, but also including the pediatric condition. This will help to provide a better overview of obesity condition effects in pediatric age, and a better identification or even prevention of the derived neuroinflammatory complications in adulthood. The main limitation of the study is the limited number of samples that characterize the cohort. In light of this, our work can be intended as a pilot-study, and future analysis can confirm our conclusions by increasing the size of the cohort and consequently including a comparable number of male and female samples in the AT-Ctrl and AT-OB groups.

## 5. Conclusions

Childhood obesity is one of the most serious public health challenges and could contribute to obesity in adulthood, leading to negative health outcomes. Transcriptomic analysis of adipose tissue of obese and normal-weight children revealed alterations in lipid, carbohydrate and fatty acid metabolic processes, along with an onset of inflammatory state. These processes, if generalized to other tissues, can contribute to an energy imbalance and the development of metabolic disorders and chronic inflammation. In the long term, these conditions could be involved in the development of neuroinflammation, resulting in neurodegenerative disorders. This work aims to give a first overview of neurodegeneration linked to the obesity condition that can start not only in old age, but can also be triggered from a pediatric age.

## Figures and Tables

**Table 1:** Clinical and metabolic features of the AT-Ctrl and AT-OB.

Clinical or Metabolic Characterization	AT-Ctrl 1	AT-Ctrl 2	AT-OB 1	AT-OB 2	AT-OB 3
Sex	male	male	male	male	female
Age	9	9	13	10	14
Weight (kg)	35	44	68	60.5	76
Height (cm)	146	144	154	148	161
Body mass index (BMI) (kg/m^2^)	16.4	21.2	28.7	27.6	29.3
Fasting blood glycemia (mg/dL) (nv < 100)	78	71	79	82	77
Tryglicerides (mg/dL) (nv ≥ 130 mg/dL if ≥ 10 years)	60	65	84	59	145
HDL-cholesterol (mg/dL) (nv < 40 in females; nv < 50 in males)	60	55	33	48	40
Triglycerides/HDL-cholesterol ratio (nv < 2.2)	1	1.1	2.5	1.2	3.6
Triglyceride–glucose index (nv < 7.88)	7.7	7.7	8.14	7.7	8.6

Triglyceride–glucose index was calculated as ln(fasting triglycerides (mg/dL) × fasting plasma glucose (mg/dL)/2), and it was assumed as a surrogate of insulin resistance. “nv” indicates the normal value for the different features.

**Table 2:** Differentially expressed genes (DEGs) up and downregulated involved in “Fatty acid metabolic process”, “Lipid metabolic process” and “Carbohydrate metabolic process”.

Biological Process	Upregulated DEGs	Fold Change	Downregulated DEGs	Fold Change
Fatty acid metabolic process	*ABCD4*	0.72	*ACAT1*	−1.13
	*ACOT8*	1.44	*ACSL1*	−1.93
	*ACSF3*	1.48	*AKR1C4*	−3.73
	*CPT1A*	0.62	*CRAT*	−0.28
	*CRYL1*	0.69	*DBI*	−1.85
	*GSTM1*	7.35	*DECR1*	−1.61
	*TNXB*	1.24	*ETFDH*	−1.15
			*HADHA*	−0.67
			*HSD17B4*	−1.14
			*PPARG*	−1.2
			*PRKAA2*	−5.69
			*SNCA*	−0.81
Lipid metabolic process	*ABCD4*	0.72	*ACAT1*	−1.13
	*ACOT8*	1.44	*ACOT13*	−1.87
	*ACSF3*	1.48	*ACSL1*	−1.93
	*AGPAT1*	0.56	*AKR1C4*	−3.73
	*ARSA*	1.82	*ATF2*	−0.76
	*CPNE1*	1.05	*CERT1*	−0.54
	*CPT1A*	0.62	*CRAT*	−0.28
	*CRYL1*	0.69	*DBI*	−1.85
	*CSF1R*	1.06	*DECR1*	−1.61
	*CYP27A1*	1.89	*DHRS4*	−0.91
	*DGKD*	0.59	*DPM1*	−0.68
	*FDXR*	4.13	*ETFDH*	−1.15
	*GGT7*	0.94	*FGF2*	−0.81
	*GSTM1*	7.35	*HADHA*	−0.67
	*HSPG2*	0.72	*HSD17B4*	−1.14
	*ITGB8*	2.50	*INSIG1*	−2.00
	*LPCAT1*	0.73	*LDAH*	−0.75
	*LRP10*	1.13	*MBTPS1*	−0.54
	*LRP2*	21.33	*MTMR12*	−0.77
	*OSBPL3*	1.13	*MTMR6*	−0.53
	*PDGFRB*	1.04	*OSBPL1A*	−0.49
	*PGAP3*	1.82	*PAFAH1B2*	−0.57
	*PIGO*	0.91	*PIK3C3*	−0.90
	*PIP5K1C*	1.21	*PPARG*	−1.20
	*PLCD1*	0.93	*PRKAA2*	−5.69
	*PLCG2*	0.85	*PTPN11*	−1.01
	*PLTP*	1.81	*SNCA*	−0.81
	*PNPLA6*	1.14		
	*PNPLA7*	2.11		
	*PRKD2*	1.18		
	*SPTLC2*	0.65		
	*TNXB*	1.24		
Carbohydrate metabolic process	*AMDHD2*	1.58	*HK1*	−0.79
	*BRAT1*	2.01	*ST6GALNAC1*	−3.85
	*CPT1A*	0.62	*UGP2*	−0.81
	*GAA*	1.23		
	*MAN1B1*	1.06		
	*SPATA20*	0.68		

DEGs involved in “Fatty acid metabolic process”, “Lipid metabolic process” and “Carbohydrate metabolic process” with fold change associated. Fold change was calculated as log_2_ (AT-OB/AT-Ctrl). In this line, the upregulated genes were more expressed in AT-OB, while downregulated genes were more expressed in AT-Ctrl. All the values are rounded to the second decimal digit.

**Table 3:** Differentially expressed genes (DEGs) up and downregulated involved in the “Regulation of fatty acid metabolic process”, “Regulation of lipid metabolic process” and “Regulation of carbohydrate metabolic process”.

Biological Process		Upregulated DEGs	Fold Change	Downregulated DEGs	Fold Change
Fatty acid metabolic process	Positive regulation	*CPT1A*	0.62	*PPARG*	−1.20
	Negative regulation			*INSIG1*	−2.00
				*PIBF1*	−0.46
				*SIRT4*	−3.97
Lipid metabolic process	Positive regulation	*BMP6*	1.71	*FGF2*	−0.81
		*CPT1A*	0.62	*PPARG*	−1.20
		*PDGFRB*	1.04	*SIRT4*	−3.97
		*PTK2B*	1.27	*SORBS1*	−1.36
	Negative regulation	*LPCAT1*	0.73	*HCAR1*	−2.38
				*INSIG1*	−2.00
				*PDE3B*	−1.53
				*PIBF1*	−0.46
				*SIRT4*	−3.97
				*SOD1*	−1.21
Carbohydrate metabolic process	Positive regulation			*ARPP19*	−1.07
				*IGF1*	−0.95
				*PRKAA2*	−5.69
				*PRXL2C*	−0.95
				*SNCA*	−0.81
				*SORBS1*	−1.36
	Negative regulation	*HDAC4*	1.11	*EP300*	−0.19
		*MST1*	2.91		

DEGs involved in the “Regulation of fatty acid metabolic process”, “Regulation of lipid metabolic process” and “Regulation of carbohydrate metabolic process” with fold change associated. Fold change was calculated as log_2_ (AT-OB/AT-Ctrl). In this line, the upregulated genes were more expressed in AT-OB while downregulated genes were more expressed in AT-Ctrl. The DEG is specified if it is involved in positive or negative regulation. All the values are rounded to the second decimal digit.

**Table 4:** Differentially expressed genes (DEGs) up and downregulated involved in the “Inflammatory response” and its regulation.

Biological Process	Upregulated DEGs	Fold Change	Downregulated DEGs	Fold Change
Inflammatory response	*AFAP1L2*	1.08	*AP3B1*	−0.51
	*BMP6*	1.71	*ASS1*	−0.73
	*CARD8*	0.74	*HK1*	−0.79
	*CD14*	1.94	*IFNGR1*	−0.96
	*CIITA*	1.28	*MS4A2*	−3.52
	*CSF1R*	1.06	*SNCA*	−0.81
	*CYBA*	0.94		
	*EPHA2*	2.26		
	*FFAR3*	3.47		
	*GRN*	0.78		
	*GSDMD*	1.44		
	*HDAC4*	1.11		
	*HNRNPA0*	0.98		
	*HSPG2*	0.72		
	*IGFBP4*	1.95		
	*IKBKB*	0.91		
	*KDM6B*	1.84		
	*LGALS9*	0.71		
	*LOXL3*	1.07		
	*MFHAS1*	1.44		
	*NFATC4*	2.05		
	*NFKBID*	3.03		
	*NLRP1*	1.71		
	*PTGDR*	1.41		
	*RPS6KA4*	1.84		
	*SIGLEC1*	1.30		
	*SMO*	2.17		
	*STAB1*	1.50		
	*TCIRG1*	1.67		
	*THEMIS2*	2.11		
	*TICAM1*	1.54		
Positive regulation of inflammatory response	*FFAR3*	3.47	*CLOCK*	−1.14
	*GPSM3*	2.80	*KARS1*	−0.62
	*GRN*	0.78	*SNCA*	−0.81
	*HLA−E*	1.52		
	*NLRP1*	1.71		
	*NLRP12*	3.16		
	*PLCG2*	0.85		
	*RPS19*	0.92		
Negative regulation of inflammatory response	*GRN*	0.78	*IGF1*	−0.95
	*MAPK7*	1.16	*PPARG*	−1.20
	*MFHAS1*	1.44	*SOD1*	−1.21
	*NLRP12*	3.16		
	*RPS19*	0.92		

DEGs involved in the “Inflammatory response” and its regulation with fold change associated. Fold change was calculated as log_2_ (AT-OB/AT-Ctrl). In this line, the upregulated genes were more expressed in AT-OB while downregulated genes were more expressed in AT-Ctrl. The DEG is specified if it is involved in positive or negative regulation. All the values are rounded to the second decimal digit.

## Data Availability

The data presented in this study are openly available in the NCBI Sequence Read Archive at BioProject accession number PRJNA839580.

## Supplementary Materials

The following supporting information can be downloaded at: https://www.mdpi.com/article/10.3390/biomedicines10081953/s1, Figure S1: PCA with AT-Ctrl samples and AT-OB samples; Table S1: Statistical scores for reads about each sample; Table S2: DEGs upregulated in AT-OB samples; Table S3: DEGs downregulated in AT-OB samples.
