# Supplementary material for: Does Childhood Obesity Trigger Neuroinflammation?
Source: Biomedicines. 2022 Aug 11;10(8):1953. doi: 10.3390/biomedicines10081953 (PMC9405861; doi:10.3390/biomedicines10081953)
Supplement: Supplementary file 1 [file biomedicines-10-01953-s001.zip › Figure S1.pdf]

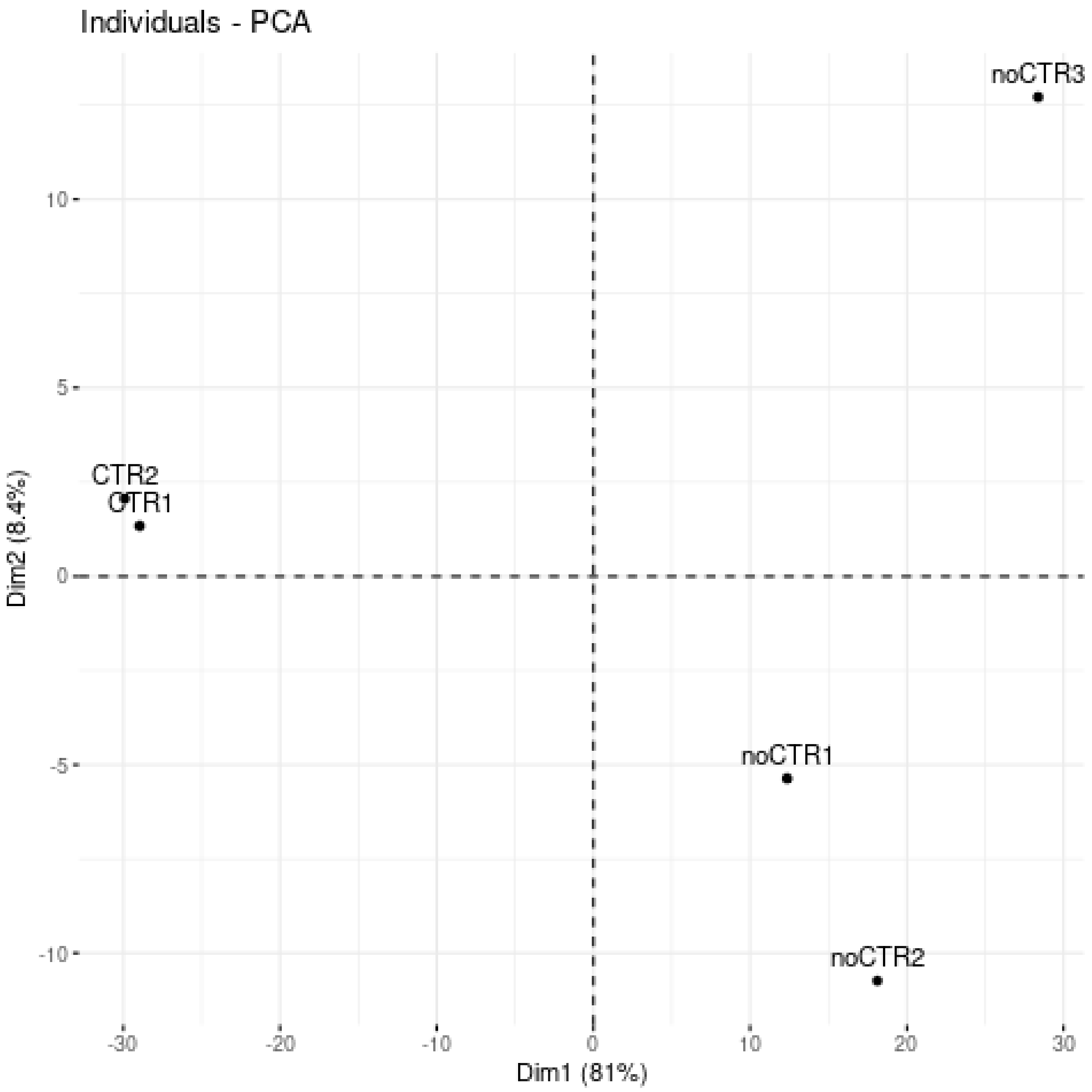

Figure S1. PCA with AT-Ctrl samples (CTR1, CTR2) and AT-OB samples (noCTR1, noCTR2, noCTR3). In X axis is reported the dimension that define the 81% of variance and in Y axis is reported the dimension that define 8.4% of variance among samples. Dim1 is quite able to discriminate AT-Ctrl and AT-OB.
