# Supplementary material for: Does Childhood Obesity Trigger Neuroinflammation?
Source: Biomedicines. 2022 Aug 11;10(8):1953. doi: 10.3390/biomedicines10081953 (PMC9405861; doi:10.3390/biomedicines10081953)
Supplement: Supplementary file 1 [file biomedicines-10-01953-s001.zip › Table S1.pdf]

Table S1. Statistical scores for each sample in AT-Ctrl or AT-OB.

	CTR1	CTR2	noCTR1	noCTR2	noCTR3
**Number of input reads**	4729009	4845249	3630402	3854851	7110689
**Average read length**	294bp	343bp	307bp	298bp	289bp
**Uniquely mapped reads number (%)**	3944291 (84,41%)	4212336 (86.94%)	2917407 (80.36%)	3018185 (78.30%)	5412256 (76.11%)
**Average depth**	1.77196	1.92041	1.34209	1.57603	7.18229

Table shows the information about number of input reads, average read length, uniquely mapped reads number and average depth for each sample. CTR1 and CTR2 are the control samples (AT-Ctrl) while noCTR1, noCTR2 and noCTR3 are the obese samples (AT-OB).
